# A noradrenergic lesion aggravates the effects of systemic inflammation on the hippocampus of aged rats

**DOI:** 10.1371/journal.pone.0189821

**Published:** 2017-12-19

**Authors:** Krishna L. Bharani, Rebecca Derex, Ann-Charlotte Granholm, Aurélie Ledreux

**Affiliations:** 1 Department of Neurosciences, Medical University of South Carolina, BSB, Charleston, SC, United States of America; 2 Knoebel Institute for Healthy Aging, University of Denver, Denver, CO, United States of America; Max Delbruck Centrum fur Molekulare Medizin Berlin Buch, GERMANY

## Abstract

Neuroinflammation is potentiated by early degeneration of the locus coeruleus noradrenergic pathway (LC-NE) commonly seen in aging-related neurodegenerative diseases such as Alzheimer’s disease and Parkinson’s disease. In animal models, lipopolysaccharide (LPS) induces strong peripheral immune responses that can cause cognitive changes secondary to neuroinflammation. The influence of the peripheral immune response on cognition might be exacerbated by LC-NE degeneration, but this has not been well characterized previously. In this study, we investigated how systemic inflammation affects neuroinflammation and cognition in aged rats that have had either normal or damaged LC-NE transmitter systems. Rats were first exposed to the selective noradrenergic (NE) neurotoxin N-(2-chloroethyl)-N-ethyl-2-bromobenzylamine (DSP4) to induce degeneration of central NE pathways. Two weeks later, the rats received a low dose of LPS. This resulted in 3 treatment groups (Control, LPS-, and DSP4+LPS-treated rats) studied at 4 hours (short-term subgroup) and 7 days (long-term subgroup) following the LPS injection. DSP4+LPS-treated rats exhibited increased serum levels of several pro-inflammatory cytokines, increased astroglial and microglial activation in the hippocampus, and poorer performance in the novel object recognition task (NORT) compared to controls and LPS-treated rats. Additionally, serum and brain tissue levels of brain-derived neurotrophic factor (BDNF) were modulated over time in the DSP4+LPS group compared to the other two groups. Specifically, DSP4+LPS-treated rats in the short-term subgroup had lower hippocampal BDNF levels (~25%) than controls and LPS-treated rats, which negatively correlated with hippocampal astrogliosis and positively correlated with hippocampal IL-1β levels. Serum and hippocampal BDNF levels in the DSP4+LPS-treated rats in the long-term subgroup returned to levels similar to the control group. These results show that systemic inflammation in LC-NE-lesioned aged rats promotes an exacerbated systemic and central inflammatory response compared to LC-NE-intact rats and alters BDNF levels, indicating the important role of this neurotransmitter system in response to neuroinflammation.

## Introduction

Aging and aging-related disorders such as Alzheimer’s disease (AD) and Parkinson’s disease (PD) are characterized by a subclinical chronic inflammatory status [1–4] and by increased pro-inflammatory markers in the brain [5]. Additionally, existing neuropathology can be exacerbated by systemic inflammation via a spread of pro-inflammatory cytokines across the blood-brain barrier (BBB) and subsequent activation of microglial cells in the brain [4,6–8]. Systemic inflammation induced by the bacterial endotoxin lipopolysaccharide (LPS) is well-documented to cause neuroinflammation in animal models [9,10] despite the fact that the endotoxin does not readily cross the BBB [11,12]. Instead, circulating pro-inflammatory cytokines induced by systemic LPS cross the BBB, activate microglia, and directly inhibit crucial transcription factors in hippocampal neurons to induce neurodegeneration secondary to neuroinflammation [4,6–8,13,14]. Several clinical and epidemiological studies suggest that pro-inflammatory cytokines can increase the susceptibility to cognitive impairment in older adults [2,8,15–18]. Accordingly, factors that further damage the BBB and increase its permeability, such as aging and noradrenergic degeneration [19,20], can exacerbate the detrimental neuronal effect of LPS-induced systemic inflammation.

Noradrenergic degeneration has been shown to occur early in AD progression and is found in amnestic mild cognitive impairment patients [21]. Understanding effects of systemic inflammation in an aged model with locus coeruleus noradrenergic (LC-NE) dysfunction will be essential for developing therapies to mitigate cognitive decline in older adults, but combined examination of LPS and LC-NE degeneration has not been undertaken to date. LC-NE neurons have extensive innervations of cortical and subcortical brain regions and modulate cognitive functions including memory and attention [22]. In particular, the LC-NE influences cognitive flexibility, working memory, and attentional processes [23]. LC-NE neurons have also been shown to degenerate in normal aging, and, to a greater extent, in both AD and PD early in the disease process [24–26]. In addition to its role as a neurotransmitter, NE also has potent anti-inflammatory effects via adrenergic receptors on astrocytes and microglia to suppress the expression of pro-inflammatory cytokines and chemokines [23,27,28]. Accordingly, LC-NE degeneration leads to the loss of the anti-inflammatory state in the brain [23,29,30], and elevated neuroinflammation due to LC-NE pathway degeneration has been linked to early neuronal dysfunction and aggravated AD pathophysiology including increased amyloid accumulation in AD mouse models [28]. Because of adrenoreceptors located on endothelial cells in the brain, the degeneration of LC-NE innervation is implicated in the disruption of tight junction assembly [19] and increased BBB permeability [20], which is likely to make the central nervous system more susceptible to systemic insults. Although these findings individually implicate LC-NE pathway degeneration and systemic inflammation in pathologies of aging, the interaction between LC-NE degeneration and systemic inflammation in the aged brain remains to be explored.

Systemic inflammation induced by LPS may also affect neurotrophic support. For example, brain-derived neurotrophic factor (BDNF) is broadly important for regulating neuronal growth, differentiation, and survival and is necessary for neuronal plasticity along with counteracting amyloid toxicity in cell culture [31–33]. Systemic inflammation was shown to reduce BDNF gene expression in certain areas of the rodent brain [34], and pro-inflammatory cytokines interfere with BDNF’s neuroprotective effects in rat brain tissue cultures [35,36]. Inflammatory disruption of BDNF synthesis and function can lead to dysfunctions in rat hippocampal-dependent memory [14,37]. LC-NE activity and BDNF seem to have an intricate relationship. Indeed, exogenous BDNF infusion into the frontal cortex protects against age-related LC-NE degeneration in rats [38]. Because LC-NE activity regulates the expression of BDNF [39,40], we wanted to explore whether a combined systemic inflammation and LC-NE degeneration would affect BDNF levels in serum or in brain tissue in aged rats.

The neurotoxin DSP4 (N-(2-chloro ethyl)-N-ehtyl-bromo-benzyl amine) readily crosses the BBB to cause selective degeneration of the rat LC-NE system by first inhibiting the noradrenaline transporter, depleting intracellular NE, and finally inducing degeneration of noradrenergic terminals. Although peripheral administration of DSP4 decreases NE levels in the peripheral sympathetic system, this effect is temporary and is resolved within 1 week (for review, see [41]). Our lab has previously shown that LC-NE degeneration induced by DSP4 significantly promotes neuroinflammation and behavioral deficits in the Ts65Dn mouse model of Down syndrome, but has no neuroinflammatory and minimal behavioral effects on normosomic mice [23]. Similarly, DSP4 treatment on rats has had minimal to no effect on behavioral performance in the open field test [42], elevated plus maze [43], water maze [44], and Cogitat holeboard paradigm [45]. Thus, the overall purpose of the current study is to examine whether a DSP4-induced LC-NE lesion would potentiate neuroinflammation and behavioral impairment specifically in aged rats subjected to LPS-induced systemic inflammation. We hypothesized that aged rats with pronounced LC-NE degeneration would develop an exacerbated response to LPS-induced systemic inflammation in terms of inflammatory markers, glial activation, and neuronal deterioration and that this would be related to a reduction in BDNF expression and reduced performance in a novel object recognition task.

## Materials and methods

### Animals

Twenty-month-old male Fischer 344 (F344) rats (weighing 434 ± 34 g) from the aging colony of National Institute on Aging (NIA) at Charles River were pair-housed in an AAALAC accredited animal care facility at the Medical University of South Carolina (MUSC). All animals were maintained on a 12-h light/dark cycle according to NIA guidelines for aged rats and received food and water *ad libitum*. All experimental procedures were approved by the Institutional Animal Care and Use Committee (IACUC) at MUSC and complied with NIH guidelines.

### Treatment

Treatment groups consisted of 16 double-saline-injected rats (Ctrl), 18 saline- and lipopolysaccharide- (LPS, from *Escherichia coli*, serotype O55:B5, Sigma-Aldrich) injected rats (LPS), and 19 DSP4- [N-(2-chloroethyl)-N-ethyl-2-bromobenzylamine, Sigma-Aldrich] and LPS-injected rats (DSP4+LPS). Rats first received one dose of DSP4 (25 mg/kg, dissolved in sterile saline 0.9%, i.p.), known to be capable of crossing the BBB and toxic to norepinephrine (NE) neurons [19,23,41] or saline (i.p.). Two weeks following the DSP4 or saline injection, rats were injected with LPS (0.75 mg/kg, dissolved in sterile saline 0.9%) or saline (i.p.). After 4 hours, a subgroup of rats was sacrificed by an overdose of isoflurane (short-term subgroup). The remaining subgroup of rats underwent behavioral assessment 7 days after LPS injection and was then sacrificed (long-term subgroup).

### Novel object recognition task (NORT)

Seven days following the LPS injection, the long-term subgroup of rats completed a three-day novel object recognition task. On Day 1, rats were habituated to an empty circular testing arena (80 cm wide) for 5 minutes. On Day 2, rats were first exposed to two identical objects (A, A) for a 5-minute period (Trial 1). After a 90-minute break, rats were exposed to one familiar and one novel object (A, B) and rats were free to explore for another 5 minutes (Trial 2). On Day 3, one of the objects was replaced with a new object (A, C), and rats explored for 5 minutes (Trial 3). After a 90-minute break, the novel object (C) was moved 90^o^ clockwise, and rats were placed in the testing arena for another 5 minutes (Trial 4). Each trial was recorded and analyzed through an automated tracking system (Panlab SMART v3.0, Harvard Apparatus, USA).

### Blood and brain collection

Rats were anesthetized deeply with isoflurane, and blood was collected by cardiac puncture into BD Vacutainer® SST tubes. Blood was allowed to clot for 1 hour at room temperature before being centrifuged for 20 minutes at 1,500 x g. Serum was aliquoted and stored in -80°C freezer until further analysis. The right frontal cortex and right hippocampus were snap frozen on dry ice and stored at -80°C until homogenization.

### Preparation of brain homogenates

Brain homogenates were prepared in homogenization buffer (20 mM Tris-HCl, 137 mM NaCl, 2.7 mM KCl, 8.1 mM Na_2_HPO_4_, 1.5 mM KH_2_PO_4_, 10% glycerol, 1% NP-40) with cOmplete™ Protease Inhibitor Cocktail (Roche Diagnostics, Ltd., Mannheim, Germany) for quantification of BDNF or IL-1β with commercial ELISA kits. Briefly, 0.02–0.05 g of brain tissue were homogenized using a mechanical tissue grinder in 1:10 w/v homogenization buffer for 20 seconds. Samples were then incubated on ice for 30 minutes before the suspension was centrifuged at 10,000 x g for 20 minutes at 4°C. The pellet was re-homogenized with 1:5 w/v homogenization buffer for 20 seconds, incubated on ice for 30 minutes, and centrifuged at 10,000 x g for 20 minutes at 4°C. The supernatants were combined, aliquoted, and stored in a -80°C freezer prior to use.

### Quantification of cytokines and chemokines

Serum levels of cytokines (IL-1β, IL-6, IL-10, IL-17A, TNFα, and IFNγ) and chemokine (IP-10) were determined using the Multiplexing LASER Bead Assay (Eve Technologies, Canada). Hippocampal levels of the pro-inflammatory cytokine IL-1β were quantified using a commercial ELISA kit (R&D Systems, Minneapolis, USA) following manufacturer’s instructions. IL-1β levels in tissue homogenates are expressed per mg of protein as determined by a BCA protein assay (Thermo Scientific, Rockford, IL, USA).

### Quantification of BDNF

BDNF levels in serum samples (diluted 1:10 with manufacturer’s provided diluent RD6P) and brain homogenates (diluted 1:2 with RD6P) were measured in duplicates by ELISA using the human BDNF Quantikine kit (R&D Systems, Minneapolis, USA), according to the manufacturer’s instructions. BDNF levels in homogenates are expressed per mg of protein as determined by a BCA protein assay (Thermo Scientific, Rockford, IL, USA).

### Immunohistochemistry

Rats were anesthetized deeply with isoflurane gas (Novaplus) and the brain was rapidly removed and dissected. The left hemisphere was fixed in 4% paraformaldehyde for 48 hours and transferred to 30% sucrose in phosphate-buffered saline (PBS) at 4°C. Sections of hippocampus (40 μm) were sectioned using a cryostat (Microm) and processed for immunohistochemistry as previously published [46,47] using the following antibodies: TH (tyrosine hydroxylase, Abcam, 1:250), GFAP (Glial fibrillary acidic protein, Dako, 1:100) and Iba1 (ionized calcium-binding adapter molecule 1, Wako, 1:500). Briefly, free-floating sections were washed 4 times in TBS (Tris-buffered saline 0.01M, pH = 7.4) and then blocked for 1 hour at room temperature with 10% normal donkey serum in TBS-T (TBS with 0.2% Triton-X 100). Sections were incubated with primary antibodies for 24 hours at 4°C, washed with TBS, and then incubated with secondary antibodies (Alexa 594 or Alexa 488, Life Technologies, 1:250) directed against the appropriate species for 1 hour at room temperature. Sections were washed with TBS, mounted on glass slides, air-dried, and cover-slipped with ProLong Gold antifade reagent (Molecular Probes). Photomicrographs were generated using a Nikon Eclipse 80i microscope (Nikon Instruments, Inc., Melville, NY) equipped with a QImaging Fast 1394 digital camera (QImaging, Surrey, Canada).

### Densitometry

**Fig 1:**
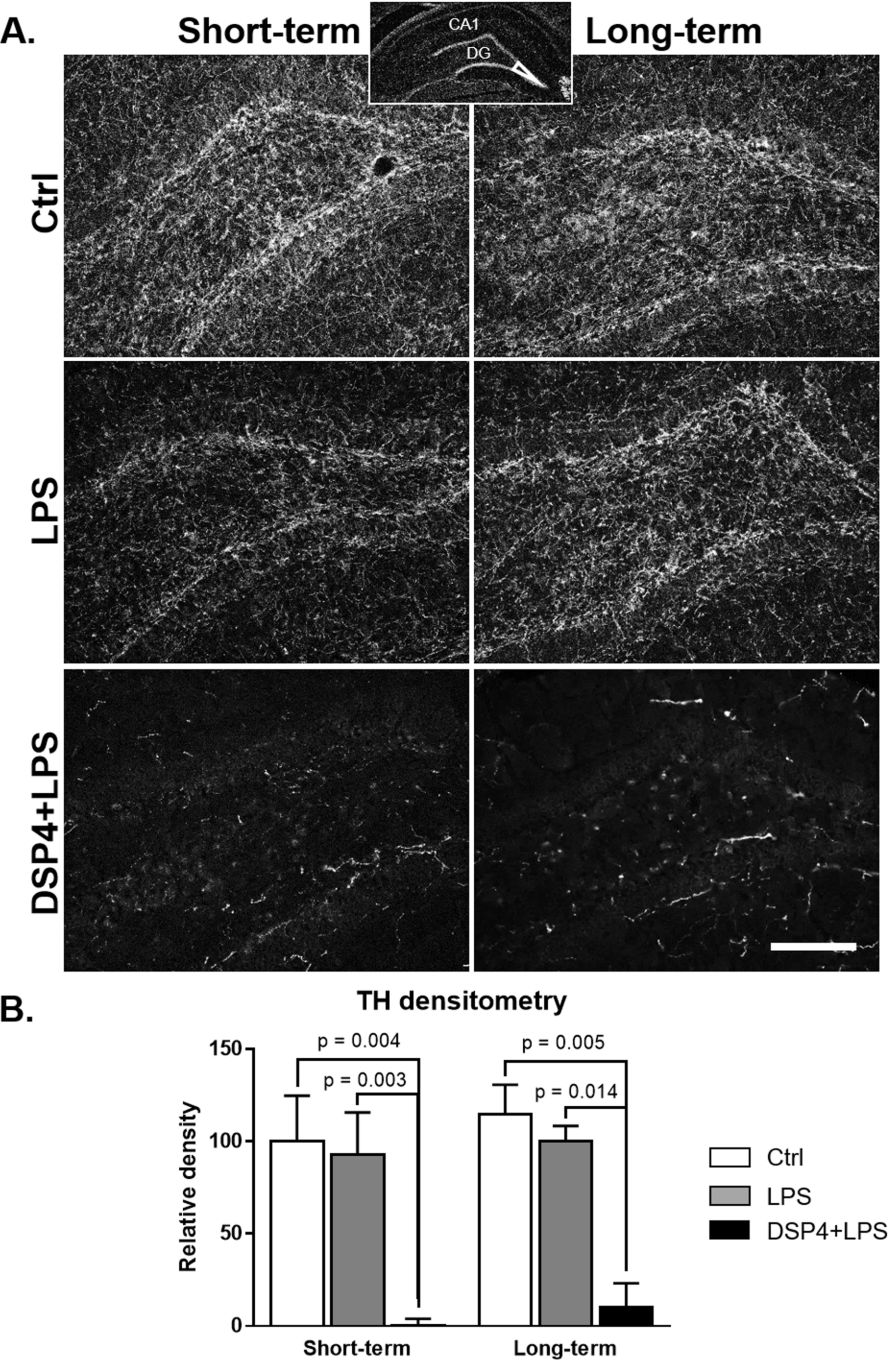
Hippocampal TH immunofluorescence. TH immunofluorescence staining shown in the hippocampal dentate gyrus (A) and densitometry (B; expressed as percent of the Ctrl group) in the short-term and long-term subgroups. The histograms demonstrate a significant reduction in TH-positive neurites in the DSP4-lesioned group, both short- and long-term, compared to the other two groups demonstrating a successful administration of the NE toxin DSP4. Densitometry confirmed observed changes, with highly significant reductions in TH density in both DSP4 groups and no changes observed in either Ctrl or LPS groups. Scale bar represents 250 μm.

Semiquantitative densitometric measurements were performed in Fiji (version 1.51h, http://imagej.nih.gov/ij) [48] using the region of interest (ROI) feature. Measurements were performed blind by one experimenter and are reported as an average of 3–4 sections per brain. Immunofluorescence staining density was obtained when background staining was subtracted from ROI staining intensities for each section. For TH and GFAP densitometry measurements, the ROI included the tip of the dentate gyrus of the hippocampus, as shown in the insert at the top of Fig 1. For Iba1 densitometry measurements, the ROI included a portion of the *stratum radiatum*, just below the *stratum pyramidale* in the CA1 of hippocampus.

### Statistical analysis

Data were reported as a mean ± standard error of the mean (SEM). Grubb’s method was used to check for outliers. Two-way ANOVA (treatment x time) were used to detect significant interactions between treatment and time after injection on relevant measurements. Tukey’s *post hoc* multiple comparison tests were used to explore, within each exposure time, which groups were significantly different. Behavior results were analyzed using one-way ANOVA with Tukey’s *post hoc* multiple comparison tests to detect changes between the 3 treatment groups. Pearson’s correlations were used when assessing relations between two variables. Statistical significance was set at p < 0.05. GraphPad Prism version 6.0 (GraphPad Software, Inc., La Jolla, CA) was used for all statistical analyses.

## Results

### DSP4 treatment caused degeneration of NE fibers in the hippocampus

As expected based on previous work [23], DSP4 lesions gave rise to a loss of tyrosine hydroxylase (TH) immunoreactivity in the hippocampus (Fig 1A). A two-way ANOVA analysis confirmed a significant effect of the treatment on TH immunoreactive fibers in the dentate gyrus (F_2,15_ = 18.52, p < 0.0001). Subsequent Tukey’s *post hoc* tests showed that DSP4+LPS-treated rats had significantly reduced TH immunoreactivity compared to the Control group (short-term: p = 0.004, long-term: p = 0.005) and LPS-treated rats (short-term: p = 0.003, long-term: p = 0.014; Fig 1B), thus demonstrating the loss of NE fibers resulting specifically from the DSP4 treatment with no effect observed from LPS alone.

### Astroglial and microglial activity was elevated in the hippocampus after DSP4+LPS treatment

**Fig 2:**
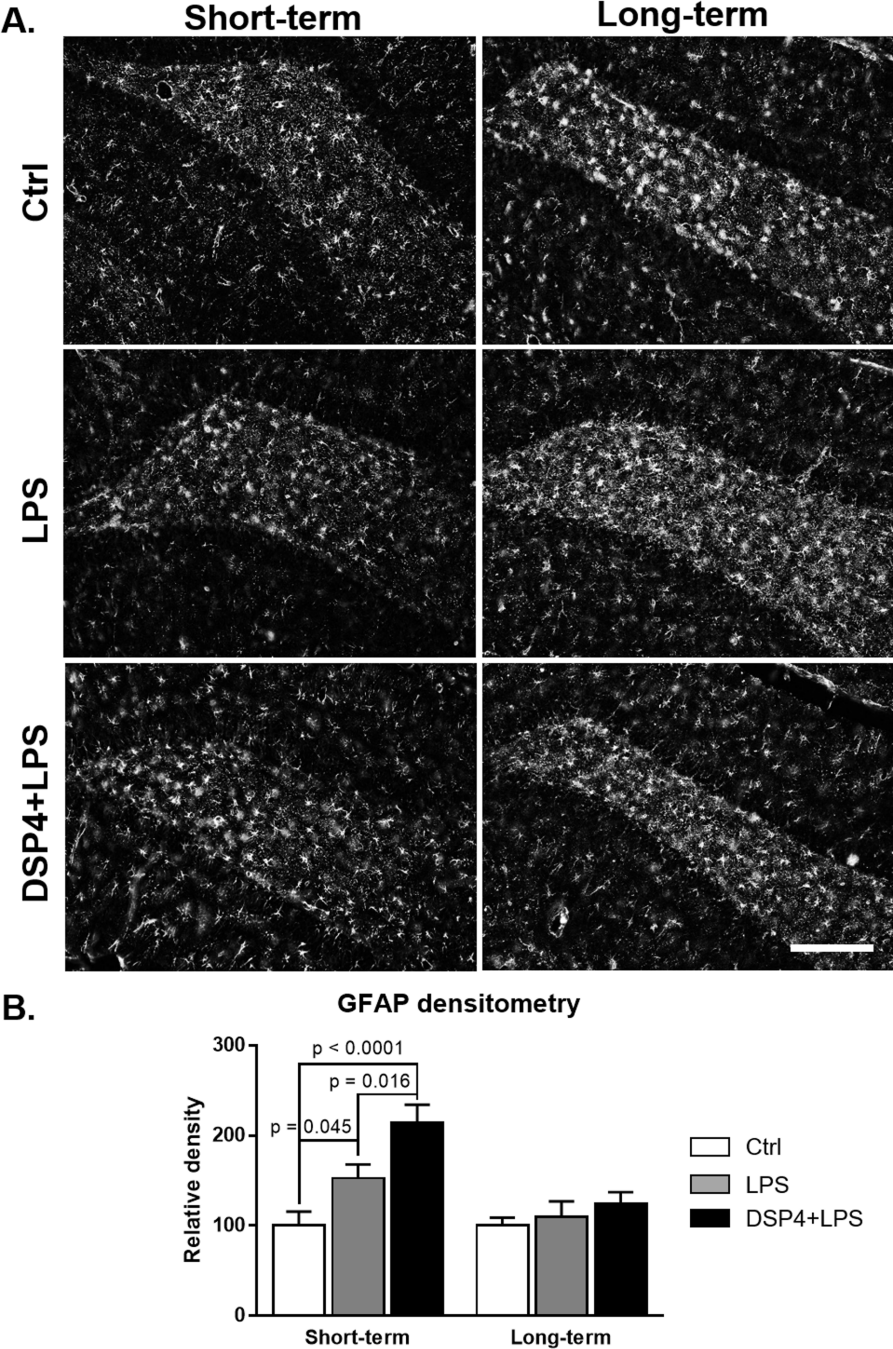
Hippocampal GFAP immunofluorescence. GFAP-immunofluorescent staining in the hippocampal dentate gyrus (A) and densitometry (B; expressed as percent of the Ctrl group) in the short-term and long-term subgroups. The histograms demonstrate a significant increase in GFAP-positive astroglia in the DSP4+LPS-treated rats in the short-term subgroup but not in the long-term subgroup. Densitometry confirmed observed changes with a highly significant increase in GFAP density in the DSP4+LPS group compared to Ctrl and LPS groups in the short-term subgroup. No significant differences were found between treatment groups in the long-term subgroup. Scale bar represents 250 μm.

Effects of DSP4 and LPS treatments on astrocytic activation in the hippocampus were assessed through glial fibrillary acidic protein (GFAP) immunoreactivity (Fig 2). Both DSP4+LPS and LPS treatments gave rise to significantly increased staining with GFAP antibody in this brain region, with the most intense increase observed in the dentate gyrus of the DSP4+LPS-treated rats in the short-term subgroup (Fig 2A). A two-way ANOVA on GFAP densitometry measurements (expressed as percent of controls) showed significant effects of the treatment (F_2,33_ = 8.92, p = 0.001) and time after LPS administration (F_1,33_ = 11.36, p = 0.002) as well as a significant interaction between treatment and time (F_2,33_ = 3.80, p = 0.033). Tukey’s *post hoc* test revealed that in the short-term subgroup, the DSP4+LPS-treated rats had significantly higher GFAP immunoreactivity than the Control (p < 0.0001) and LPS (p = 0.016, Fig 2B) groups. GFAP immunoreactivity in the LPS group was also elevated compared to the Control group (p = 0.045; see Fig 2B). Seven days following the LPS treatment, no significant difference was observed in reactive astrogliosis in the dentate gyrus between the three groups, suggesting that the astrocytic activation following LPS and DSP4+LPS treatment had subsided at this point in the long-term group.

**Fig 3:**
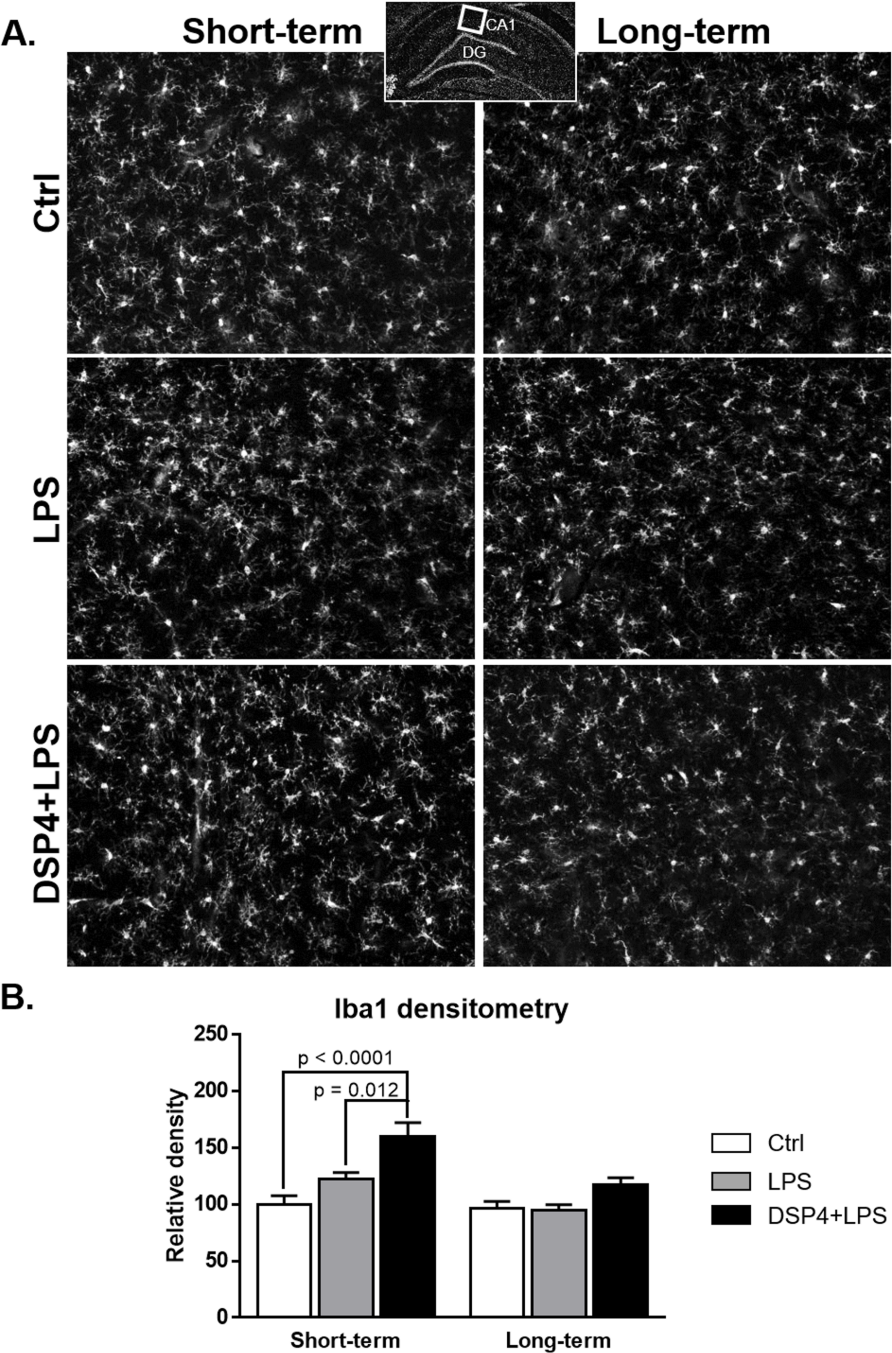
Hippocampal Iba1 immunofluorescence. Iba1-immunofluorescent staining in the CA1 (A) and densitometry (B; expressed as percent of the Ctrl group) in the short-term and long-term subgroups. Densitometry measurements demonstrate a significant increase in Iba1-microglia in the DSP4+LPS-treated rats compared to Ctrl and LPS groups in the short-term subgroup but not in the long-term subgroup. Scale bar represents 250 μm.

Microglial activation was assessed with Iba1 immunoreactivity in the hippocampus (Fig 3). Both DSP4+LPS and LPS treatments resulted in increased Iba1 immunoreactivity in the CA1 of the hippocampus in the short-term group. A two-way ANOVA on Iba1 densitometry measurements (expressed as percent of controls) showed significant effects of the treatment (F_2,32_ = 14.76, p < 0.0001) and time after LPS administration (F_1,32_ = 15.24, p = 0.0005), with Tukey’s *post hoc* tests revealing that the DSP4+LPS-treated rats had significantly higher Iba1 immunoreactivity compared to the Control (p < 0.0001) and LPS (p = 0.012, Fig 3B) groups. No significant difference was found in the long-term group.

### Serum cytokines and chemokines levels were increased in response to DSP4+LPS treatment

In order to determine whether treatment or time after the LPS injection affected peripheral inflammatory markers, two-way ANOVA followed by Tukey’s *post hoc* multiple comparison tests were conducted on serum IL-1β, IL-6, IL-10, IL-17A, IFNγ, TNFα, and IP-10 levels. A significant statistical interaction was found between the effect of treatment and time after LPS injection, as well as a main effect of treatment and a main effect of time, as shown in Table 1.

**Table 1:** Two-way ANOVA results for serum cytokines and chemokine levels.

Serum Cytokine/Chemokine	Treatment	Time	Interaction
IL-1β	F_2,43_ = 3.84, p = 0.029	F_1,43_ = 9.80, p = 0.003	F_2,43_ = 4.34, p = 0.019
IL-6	F_2,46_ = 3.94, p = 0.026	F_1,46_ = 6.23, p = 0.016	F_2,46_ = 3.93, p = 0.027
IL-10	F_2,43_ = 3.71, p = 0.033	F_1,43_ = 12.89, p = 0.001	F_2,43_ = 3.89, p = 0.028
IL-17A	F_2,47_ = 6.63, p = 0.003	F_1,47_ = 6.64, p = 0.013	F_2,47_ = 4.96, p = 0.011
IFNγ	F_2,47_ = 6.39, p = 0.004	F_1,47_ = 24.0, p < 0.0001	F_2,47_ = 7.54, p = 0.001
TNFα	F_2,46_ = 12.3, p < 0.0001	F_1,46_ = 43.7, p < 0.0001	F_2,46_ = 11.6, p < 0.0001
IP-10	F_2,46_ = 11.8, p < 0.0001	F_1,46_ = 29.8, p < 0.0001	F_2,46_ = 11.9, p < 0.0001

**Fig 4:**
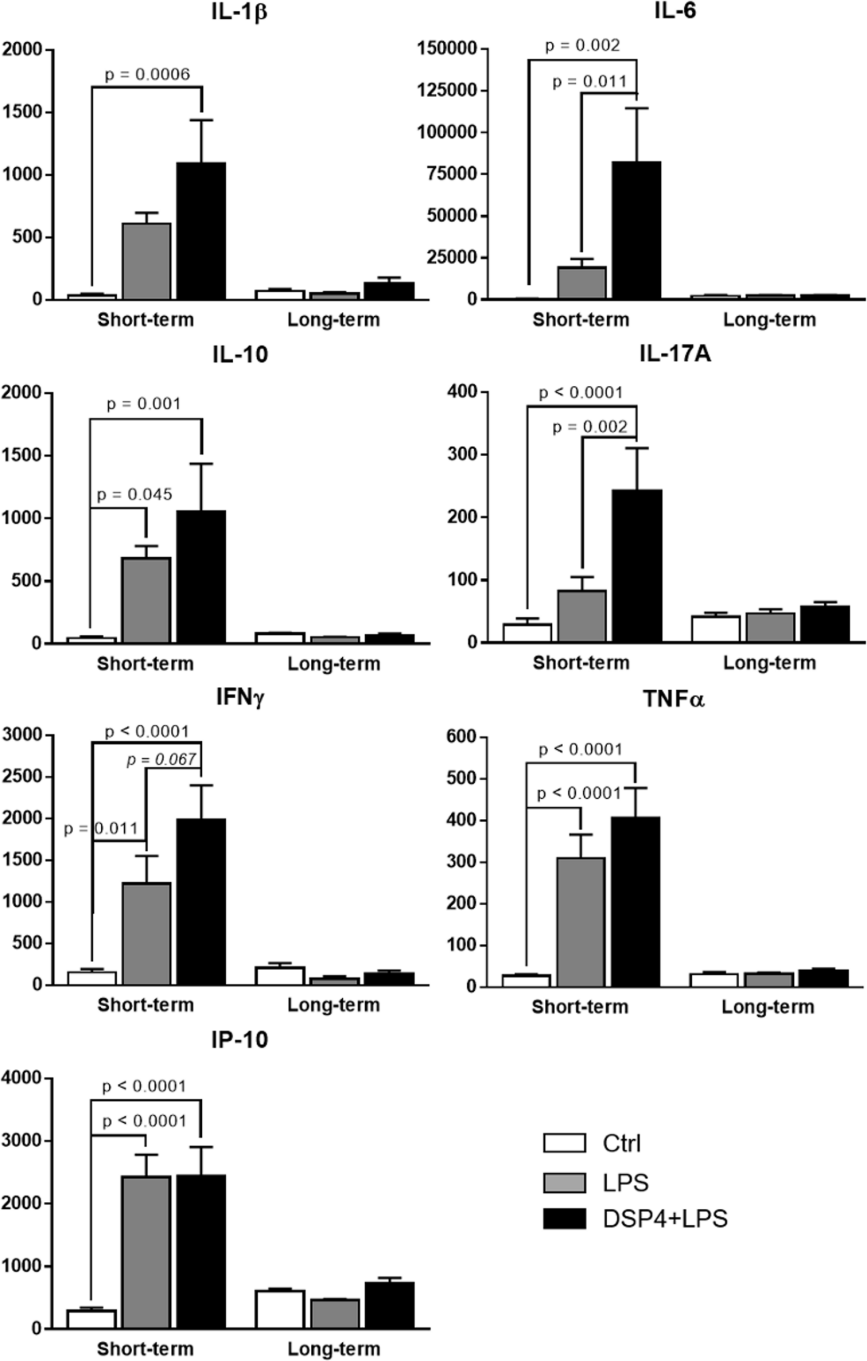
Serum cytokines/chemokine levels in the short-term and long-term subgroups for the control, LPS-, and DSP4+LPS-treated rats. All levels are expressed in pg/mL ± SEM. In the short-term subgroup, serum cytokine levels including IL-1β, IL-6, IL-10, IL-17A, TNFα, IFNγ, and the chemokine IP-10 were significantly different between the three groups. Tukey’s *post hoc* test suggest that the NE lesion caused by the DSP4 toxin exacerbated the immune response caused by the LPS challenge in the short-term, with increased cytokine levels in the DSP4+LPS group compared to the LPS group. The absence of differences in serum cytokine levels between the 3 treatment groups in the long-term suggests that the acute cytokine response to the LPS-induced systemic inflammation was resolved after 7 days.

In the short-term subgroup, Tukey’s *post hoc* multiple comparisons tests showed consistent, significant differences between the Control and DSP4+LPS groups for all the aforementioned cytokines, revealing that NE lesions due to DSP4 treatment and peripheral inflammation caused by LPS resulted in elevated serum cytokines and chemokine levels when compared to the Control group (see Fig 4 for p values). The NE lesion caused by the DSP4 toxin exacerbated the immune response caused by the LPS challenge, which increased cytokine levels in the DSP4+LPS group compared to the LPS group. However, further statistical analysis (Tukey’s *post hoc* test) revealed significant differences between these two groups only for IL-6 and IL-17A (p = 0.011 and p = 0.002, respectively) while a difference trending towards significance was found for IFNγ (p = 0.067), most likely because of the high variability observed within the 3 short-term treatment groups for this cytokine. Similarly, the LPS treatment resulted in elevated serum cytokines relative to the Control group, and Tukey’s *post hoc* multiple comparison tests showed significant differences for the cytokines TNFα, IL-10, IFNγ (p < 0.0001, p = 0.045, and p = 0.011, respectively) and chemokine IP-10 (p < 0.0001) (see Fig 4).

In the long-term subgroup, it is noteworthy that serum cytokine levels for LPS and DSP4+LPS groups were returned to levels similar to those of the Control group and were approximately one order magnitude lower when compared to the short-term subgroup (Fig 4). Overall, the absence of differences in serum cytokine levels between the 3 treatment groups 7 days after LPS treatment suggests that the cytokine and chemokine response to the LPS-induced systemic inflammation was resolved after 7 days.

### Hippocampal levels of IL-1β were transiently elevated after LPS challenge in DSP4-treated rats

**Fig 5:**
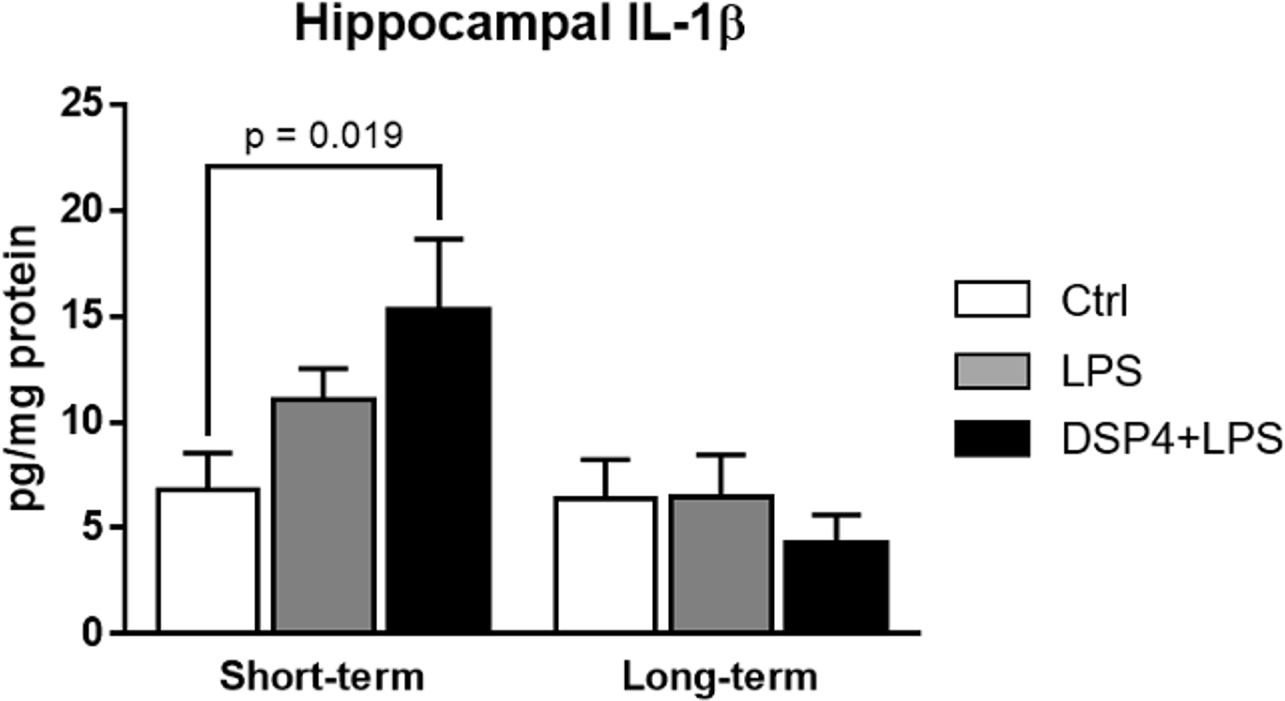
IL-1β levels in the hippocampus as determined by ELISA in short-term and long-term subgroups. Levels are expressed in pg/mg protein ± SEM. A significant increase in hippocampal IL-1β levels in DSP4+LPS-treated rats compared to the Control rats suggests an exacerbated neuroinflammatory response caused by the LPS challenge in the short-term subgroup, an effect that is not seen in the long-term subgroup.

Elevated levels of IL-1β in the hippocampus have been repeatedly shown to impair hippocampal-dependent memory by affecting the long-term potentiation [37,49–57]. In order to determine if the LPS and DSP4+LPS treatments resulted in exacerbated neuroinflammation, the levels of IL-1β were quantified by ELISA in the hippocampus of Control, LPS-treated, and DSP4+LPS-treated rats in the short-term and long-term subgroups. Our data showed that there was a significant interaction between treatment and time after LPS injection (two-way ANOVA: F_2,44_ = 3.38, p = 0.043), as wells as a significant main effect of time (F_1,44_ = 10.30, p = 0.003). In the short-term subgroup, Tukey’s multiple comparison tests showed that the DSP4+LPS-treated rats exhibited higher hippocampal IL-1β levels relative to the Control group (p = 0.019, Fig 5). In the long-term subgroup, hippocampal IL-1β levels were not affected by the treatment and were similar to levels measured in the Control group (Fig 5).

### Serum and hippocampal BDNF levels were modulated by LPS and DSP4+LPS treatments

In order to examine whether the NE lesion caused by the DSP4 toxin had further effects on BDNF levels compared to LPS effects alone, BDNF levels were assessed in serum, hippocampus, and frontal cortex by ELISA. Two-way ANOVAs were conducted to examine the effect of treatment and time after LPS injection on BDNF levels.

**Fig 6:**
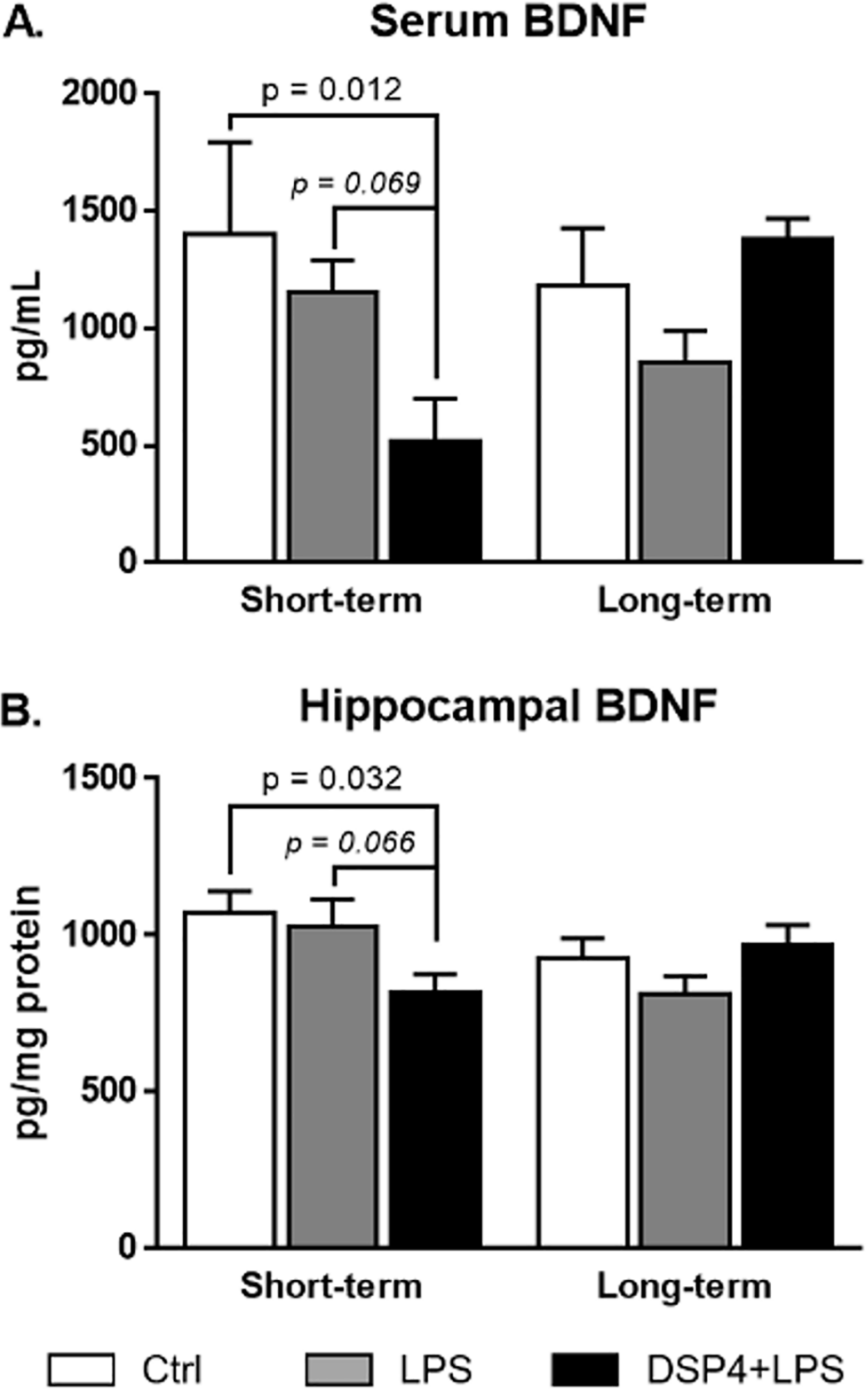
**BDNF levels in serum (A) and hippocampus (B) as determined by ELISA after short-term or long-term exposure to LPS.** Serum levels are expressed in pg/mL ± SEM and hippocampal levels are expressed in pg/mg protein ± SEM. BDNF levels in serum and hippocampus were significantly reduced in DSP4-lesioned animals after LPS challenge in the short-term subgroup compared to Ctrl.

We found a significant interaction between the effect of treatment and time after LPS injection for serum BDNF levels (F_2,45_ = 5.40, p = 0.008). In the short-term group, Tukey’s *post hoc* test showed that serum BDNF levels were significantly lower in the DSP4+LPS group relative to the Control group (p = 0.012) and were decreased relative to the LPS-treated group although it did not reach significance (p = 0.069, Fig 6A). In the long-term group, serum BDNF levels appeared increased in the DSP4+LPS group compared to the LPS, although not significantly (Tukey’s *post hoc* test: p = 0.144; see Fig 6A).

A significant interaction between treatment and time after LPS injection was found in hippocampal BDNF levels (two-way ANOVA: F_2,45_ = 4.395, p = 0.018, Fig 6B) but not in frontal cortex BDNF levels (data not shown). In the short-term group, hippocampal BDNF levels in the DSP4+LPS group were significantly lower relative to the Control group (Tukey’s *post hoc* test: p = 0.032) and trended towards significance when compared to the LPS-treated group (p = 0.066, Fig 6B). A Pearson correlation revealed a statistically significant positive correlation between BDNF levels in the hippocampus and serum (r = 0.492, p = 0.015), possibly indicating that the elevated BDNF levels in serum observed in the short-term subgroup could reflect adaptive changes in the brain. Interestingly, in the short-term group, GFAP immunoreactivity in the dentate gyrus correlated negatively with the hippocampal BDNF levels (r = -0.762, p = 0.001) and positively with hippocampal IL-1β levels (r = 0.549, p = 0.042) and Iba1 immunoreactivity (r = 0.499, p = 0.058), suggesting that lower BDNF levels and higher IL-1β levels as well as stronger microglia activation were related to increased activation of astrocytes in the hippocampus. No significant treatment effect was observed in the long-term group (Fig 6B).

### Behavior

#### Reduced spontaneous locomotion after DSP4+LPS treatment

**Fig 7:**
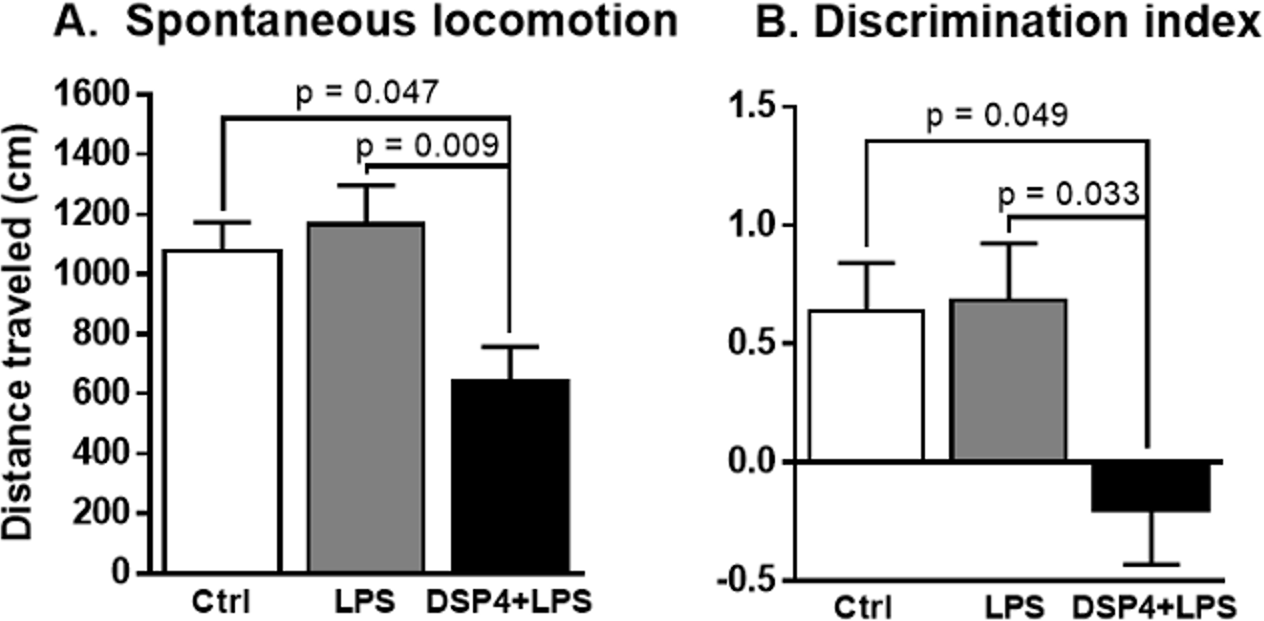
Spontaneous locomotion and discrimination index. The novel object recognition task (NORT) was administered 7 days after the LPS injection in control and DSP4-lesioned and non-lesioned rats. Spontaneous locomotion (A) indicates a reduction in movement in the DSP4-lesioned rats compared to both Ctrl and LPS groups. The discrimination index (B) was calculated as the amount of time spent exploring the novel object relative to the familiar object, divided by the total amount of time exploring both objects, and collapsed across all three testing phases. DSP4+LPS-treated rats performed worse on this task compared to the Ctrl group and the LPS group, suggesting that these rats were not able to discriminate between a familiar object and a novel object.

DSP4+LPS-treated rats exhibited a significant deficit in total distance traveled (Fig 7A). A one-way ANOVA showed significant effects of LPS and DSP4+LPS treatment (F_2,22_ = 5.998, p = 0.008) on spontaneous locomotion. Tukey’s *post hoc* analysis revealed that DSP4+LPS-treated rats performed significantly worse than LPS-treated rats (p = 0.009) and Control rats (p = 0.047), suggesting that NE lesions gave rise to reduced spontaneous movement in the aged rats.

#### Novel object recognition task

The novel object recognition task (NORT) was administered 7 days after the LPS injection in DSP4-lesioned and non-lesioned rats. NORT is indicative of exploratory behavior as well as memory function [58]. The discrimination index (DI) was calculated as the amount of time spent exploring the novel object relative to the familiar object, divided by the total amount of time exploring both objects [23,58]. In order to get a more robust estimate of the discrimination index, we collapsed the data across the 3 testing phases. A one-way ANOVA showed an overall significant difference between the treatment groups (F_2,21_ = 4.48, p = 0.024, Fig 7B). Tukey’s *post hoc* comparison revealed that the DSP4+LPS-treated group performed worse on this task compared to the Control group (p = 0.049) and the LPS group (p = 0.033), suggesting that these rats were not able to discriminate between a familiar object and a novel object. We found that the discrimination index results were negatively correlated to Iba1 immunoreactivity (r = -0.520, p = 0.027) and to GFAP immunoreactivity (r = -0.625, p = 0.017), suggesting that rats with poorer performance had increased microglial and astroglial activation.

## Discussion

Neuroinflammation induced by systemic inflammation has been implicated in the onset and progression of neurodegenerative diseases including AD, PD, and Down syndrome [2–4,10,59]. Previous studies have shown that a peripheral immune challenge in animal models of neurodegeneration exacerbates pathology and cognitive deficits and implicates the immune system’s disease modifying role [9,17,60]. The results of the present study add to this literature and show that neuroinflammation induced by systemic inflammation is exacerbated by LC-NE lesion. Specifically, in aged rats, we found that a loss of LC-NE neurons induced by the neurotoxin DSP4 combined with peripheral inflammation caused by LPS led to transient increased astrogliosis and microglial activation in the hippocampus, increased accumulation of pro-inflammatory cytokines both in the hippocampus and in serum, and reduced exploratory behavior. BDNF levels were initially reduced in both serum and hippocampus following the LPS challenge in NE-lesioned rats. Similar to previous reports on LPS-treated rats, we did not find significant differences in hippocampal BDNF levels in the LPS group alone compared to the control [61,62]. However, in the short-term subgroup, BDNF levels in the DSP4+LPS-treated rats were significantly reduced compared to Control and recovered to control levels after the long-term exposure. These results suggest that neuronal damage due to DSP4 in addition to LPS inflammation may disrupt BDNF synthesis. Thus, our results show that degeneration of the LC-NE pathway results in an exacerbated but transient systemic and neural response to a peripheral immune challenge including increased cytokine production, astrogliosis, microglial activation, and modulated BDNF levels.

We found that systemic LPS injection stimulates the release of pro-inflammatory cytokines IL-1β, IL-6, IL-17A, and TNFα in the peripheral circulation. These effects of systemic LPS administration in aged rats have also been reported by others [63–69]. *In vitro*, LPS has also been shown to induce the anti-inflammatory cytokine IL-10 in a negative feedback manner to mitigate excessive inflammation [70]. In our study, although an elevation of the aforementioned cytokines and chemokines was found in the short-term LPS-treated group relative to the Control group, this elevation was only statistically significant from control for TNFα, IL-10, IFNγ, and IP-10. However, we found a robust significant elevation of these cytokines and chemokine in the short-term DSP4+LPS group, suggesting that LC-NE degeneration by DSP4 potentiates the inflammatory response to LPS. This potentiation may be due to a transient neurotoxic effect of DSP4 (approximately seven days) on the peripheral sympathetic system in addition to the central nervous system [71,72]. In the long-term group, cytokine and chemokine levels in LPS-treated and DSP4+LPS-treated rats were returned to control levels, suggesting a resolution of inflammation after seven days. While previous studies showed increased hippocampal IL-1β levels after LPS exposure in rats [65,66], this was not the case under our experimental conditions, possibly due to differences in animal models or incubation period between LPS administration and sample collection. It is also noteworthy that the LPS dose used in our study (0.75 mg/kg) was lower than the ones used in the Fu et al. [65] (2 mg/kg) and Kawano et al. [66] (5 mg/kg) studies. However, we found that the NE lesion caused by the DSP4 toxin resulted in significantly elevated hippocampal IL-1β levels 4 hours after the LPS administration. Similar to previous reports showing that systemic inflammation by itself does not induce neurodegeneration [10], our results show that a peripheral immune challenge in aged rats resolves within seven days and does not induce long-term cognitive deficits despite inducing neuroinflammation. Our findings showed that the LPS group did not exhibit any statistically significant changes in either spontaneous locomotion or in exploratory behavior. However, we report lasting cognitive deficits in the DSP4+LPS group, potentially due to the reduction of NE levels in hippocampus caused by the DSP4 administration in the DSP4-treated rats [42]. The lack of behavioral effects in the LPS group differs from Haba et al. [73] who showed that LPS administration reduced novel object exploration in mice for at least 24 hours after injection, but this difference can be explained by our use of a different animal model and our extended time between LPS administration and cognitive testing. Indeed, Czerniawski et al. [74] report that LPS administration does not impair novel object recognition in rats 6 hours after LPS administration, suggesting that there are still conflicting results in animal models regarding short- or long-term effects of LPS on behavioral measures.

LPS influence on neuroinflammation has been largely attributed to the upregulation of cytokines that cross the BBB and interact with CNS tissue [12,75]. These LPS-induced pro-inflammatory cytokines can suppress the expression or release of BDNF [34], which is important for the survival of LC-NE neurons. Our results indicate that LPS treatment combined with DSP4 in aged rats can further reduce BDNF levels in the hippocampus, at least initially. The reduction of hippocampal BDNF protein levels was negatively correlated with the extent of neuroinflammation induced by the LPS administration. Interestingly, after the resolution of inflammation (i.e., seven days post-LPS administration), both serum and hippocampal BDNF levels in the DSP4+LPS-treated group were returned to levels similar to the control group. This reduction suggests that after the inflammatory state is resolved and pro-inflammatory cytokines are no longer suppressing BDNF, BDNF levels are upregulated both centrally and peripherally as a compensation in order to return function to normal. A similar compensatory increase in BDNF levels has been observed in other neuronal lesion models [76], lending support to the notion of BDNF increase due to lesions in the brain. It is possible that the relative reduction in BDNF levels in serum acutely after DSP4+LPS treatment result from the systemic administration of the NE neurotoxin affecting the sympathetic nervous system and therefore cause degenerative changes in BDNF in serum. As shown by Ross and Stenfors [41], NE levels in the periphery were reduced as well after systemic DSP4 lesions but recovered fully within a week of injection, which could explain why peripheral cytokines and BDNF levels are affected by the NE neurotoxin. Similar to the pro-inflammatory effects of NE degeneration in the brain [19], the peripheral sympathetic system acts to suppress inflammatory reactions in the peripheral nervous system [77]. It is therefore not unlikely that the DSP4 lesions could have given rise to sympathetic changes in the short term which would have affected the expression of BDNF and initiated peripheral inflammation, aggravated by LPS. An interaction between the peripheral and central NE transmitter systems may be important especially with aging, since studies have shown not only age-related degeneration of the LC-NE nucleus but also reduced sympathetic nervous system innervation in target tissues in the periphery with aging [78]. This interesting connection between aging effects in peripheral versus central NE afferents will be pursued in future studies. In conclusion, our results indicate that NE depletion increases susceptibility to neuroinflammation induced by systemic inflammation in aged rats. This result is relevant to the aging community where NE levels are decreased due to natural and pathology-associated degeneration of the LC-NE pathways [30,79]. An age-related reduced LC-NE function leading to an increased inflammatory reaction both in the periphery and in the brain could aggravate severe effects of immune challenges in the elderly [80]. Epidemiological studies have shown strong influence of peripheral inflammation on cognitive performance in elderly subjects [81–83]. For example, Kesse-Gyout et al. [83] suggested that a pro-inflammatory diet at midlife might be associated with subsequent lower cognitive functioning. Our findings in aged rats support this previous suggestions and also point to a complicated relationship between inflammation and NE innervation that affects both peripheral and central systems. Our findings suggest future treatment options, such as using NE-enhancing drugs that may affect not only neuronal components of the brain but may also mitigate ongoing inflammatory processes both in the brain and peripherally.
